# *Mycobacterium tuberculosis* does not inhibit NLRP1 and pyrin inflammasomes in human macrophages

**DOI:** 10.1128/spectrum.04121-25

**Published:** 2026-06-18

**Authors:** Akshaya Ganesh, Shivangi Rastogi, Volker Briken

**Affiliations:** 1 Department of Cell Biology and Molecular Genetics, University of Maryland 171289, College Park, Maryland, USA; Indian Institute of Science Bangalore, Bangalore, Karnataka, India

**Keywords:** host-pathogen interactions, inflammasome, macrophages, immunology, innate immunity, mycobacteria, microbiology

## Abstract

*Mycobacterium tuberculosis* (Mtb) has been known to evade host innate immunity by manipulating macrophage function. Interleukin-1β (IL-1β) is a cytokine secreted by macrophages as a consequence of inflammasome activation. Mtb can inhibit activation of the NLRP3 and AIM2 inflammasomes and subsequent pyroptosis. The capacity of Mtb to manipulate other types of inflammasomes is unknown. In this study, we investigated whether Mtb or the nontuberculous mycobacteria *Mycobacterium kansasii* (Mkan) and *Mycobacterium smegmatis* (Msmeg) can inhibit the NLRP1 or pyrin inflammasomes. We show that none of the mycobacteria consistently inhibit the NLRP1 inflammasome after it is activated by priming with lipopolysaccharide (LPS) and treatment with 1G244 in human macrophages. Similarly, neither Mtb nor the NTMs inhibit the pyrin inflammasome after it is activated by priming with LPS and treating with TcdB toxin from *Clostridium difficile* in human macrophages. In murine macrophages, Mkan shows inhibition only at 3 h post-infection, and Msmeg, only at 24 h post-infection, whereas no inhibition was observed at any time point for Mtb. In conclusion, our main findings are that Mtb is unable to inhibit the NLRP1 and the pyrin inflammasomes in human macrophages.

**IMPORTANCE:**

The research focuses on the interaction of mycobacteria with BMDMs and hMDMs regarding NLRP1 and pyrin inflammasome inhibition. We think that these results point to insights into the pathways of pathogen recognition in macrophages, which may have broader implications for host defense. These results are technically sound and provide new information to the field by showing that various mycobacteria do not inhibit the NLRP1 and Pyrin inflammasomes, at least under our specific experimental settings. Important limitations to the study are its narrow scope and limited mechanistic depth.

## INTRODUCTION

Intracellular pathogens are detected by oligomeric protein complexes known as the inflammasome (1). The inflammasome contains leucine-rich repeat-containing receptors (NLRs) or absent in melanoma 2-like receptors (ALRs), both of which form the pathogen or danger recognition component of the inflammasome. These molecules are often associated with the adaptor protein (ASC) and always bind to the protease, pro-caspase-1. Once activated, pro-caspase-1 cleaves itself to generate active caspase-1, which cleaves pro-IL-1β into its mature form. Caspase-1 also cleaves Gasdermin-D (GSDMD), and the generated GSDMD N-terminal fragment forms pores in the cell membrane, allowing IL-1β to exit the cell. Subsequently, NINJ1 is activated, leading to rupture of the plasma membrane and cell lysis. This overall process is classified as pyroptosis (2).

NLRP1 inflammasome is involved in the detection of *Shigella flexneri*, *Listeria monocytogenes*, and *Toxoplasma gondii,* as well as sensing viral proteases, such as pathogenic coronavirus protease 3CL (3, 4). Mutations in NLRP1 have been associated with inflammatory skin disorders and metastatic melanoma (5, 6). Humans only encode one NLRP1 gene, whereas mice have multiple paralogs of the NLRP1 gene. NLRP1b was shown to induce caspase-1 activation in response to lethal toxin (LeTx) from *Bacillus anthracis* in S129 mice (4). Serine dipeptidyl peptidases DPP8/9 can cleave the N-terminal dipeptides in limited substrates, including murine macrophages (7). Human NLRP1 detects protease cleavage by certain viral proteases, ribotoxic stress, and dsRNA binding by certain alphaviruses in epithelial cells (8). For both human and murine cells, at the baseline state, NLRP1 remains autoinhibited; however, the inhibition of DPP8/9 can induce the cleavage of NLRP1, leading to activation and subsequent pyroptosis (9, 10). Despite the differences in murine and human NLRP1 activity, all vertebrate NLRP1 proteins are bound to DPP9. Therefore, the disruption of this binding is a universal mechanism for activating the NLRP1 protein (8). NLRP1 has yet to be studied in the context of mycobacterial infection.

The pyrin protein has been implicated in several hereditary inflammatory diseases, namely Familial Mediterranean Fever. The protein is associated with the adaptor protein ASC and caspase-1 to form an inflammasome complex that can process and release IL-1β and IL-18 (11). Pyrin is primarily expressed in peripheral leukocytes of the blood. Pyrin is present at sites of increased actin polymerization and interacts with actin filaments and microtubules (12–14). Rho GTPases are proteins that exist as molecular switches for a variety of cellular processes within the cell, such as cytoskeleton organization and immune cell migration (14). Virulence factors from pathogens can downregulate the activity of RhoA, and this disruption can induce the activation of the pyrin inflammasome in murine macrophages. In human monocyte-derived macrophages and THP-1 cells, RhoA downregulation leads to IL-1β release via the NLRP3 pathway (15). Steroid hormone catabolites induce pyrin inflammasome activation via a non-canonical pathway (16).

*Mycobacterium tuberculosis* (Mtb) is a human-specific, intracellular pathogen that activates the NLRP3 inflammasome in infected macrophages (17). IL-1β has been implicated in inducing a protective immune response in the host against Mtb infection (18, 19). Mtb can, to a certain extent, also evade recognition by the NLRP3 inflammasome in murine macrophages and dendritic cells (20). The Mtb-specific phospholipid phosphatase (PtpB) cleaves the host cell membrane phosphatidylinositol-4-phosphate, reducing GSDMD-N insertion into the plasma membrane and interrupting pyroptosis and IL-1β release (21). Protein kinase F (PknF) of Mtb limits NLRP3 inflammasome activation, although the mechanism remains unclear (22). The AIM2 inflammasome is inhibited by Mtb in murine dendritic cells in the absence of NLRP3 through an IFNβ- and ESX-1-dependent manner (23). *Mycobacterium kansasii* (Mkan), an opportunistic pathogen, and *Mycobacterium smegmatis* (Msmeg), a non-pathogenic bacterium, are interesting relatives of Mtb with decreasing potential for immune evasion. The study of these related pathogens can provide insights into the behavior of Mtb and its activity. Mkan infection leads to the release of IL-1β in THP-1 macrophages via activation of the NLRP3 inflammasome (7, 24). Msmeg can induce a strong apoptosis response in comparison to more virulent mycobacteria (25). Interestingly, Msmeg activates the AIM-2 inflammasome in addition to the NLRP3 inflammasome in murine bone marrow-derived dendritic cells (23).

In this study, we investigated whether Mtb, Mkan, or Msmeg can inhibit NLRP1 or pyrin inflammasome activation. We found that none of the studied mycobacterial species inhibited the NLRP1 or pyrin inflammasomes in human macrophages. Conversely, in murine macrophages, at select time points, Mkan and Msmeg, but not Mtb, could inhibit the NLRP1 inflammasome.

## RESULTS AND DISCUSSION

### *M. smegmatis* does not inhibit the NLRP1 inflammasome in human macrophages

1G244, a specific inhibitor of DPP8/9 (26), was added to primary human monocyte-derived macrophages (hMDMs) and mouse bone marrow-derived macrophages (BMDMs) that were previously stimulated with LPS for 4 h to determine the concentrations at which IL-1β production could be observed. For subsequent studies, the 3 μM 1G244 dose was chosen because it elicited an easily detectable IL-1β amount at 3–4 h and 24 h post-treatment. This response had not reached a plateau yet, since adding 10 μM 1G244 further increased the secreted IL-1β amounts (Fig. S1).

**Fig 1:**
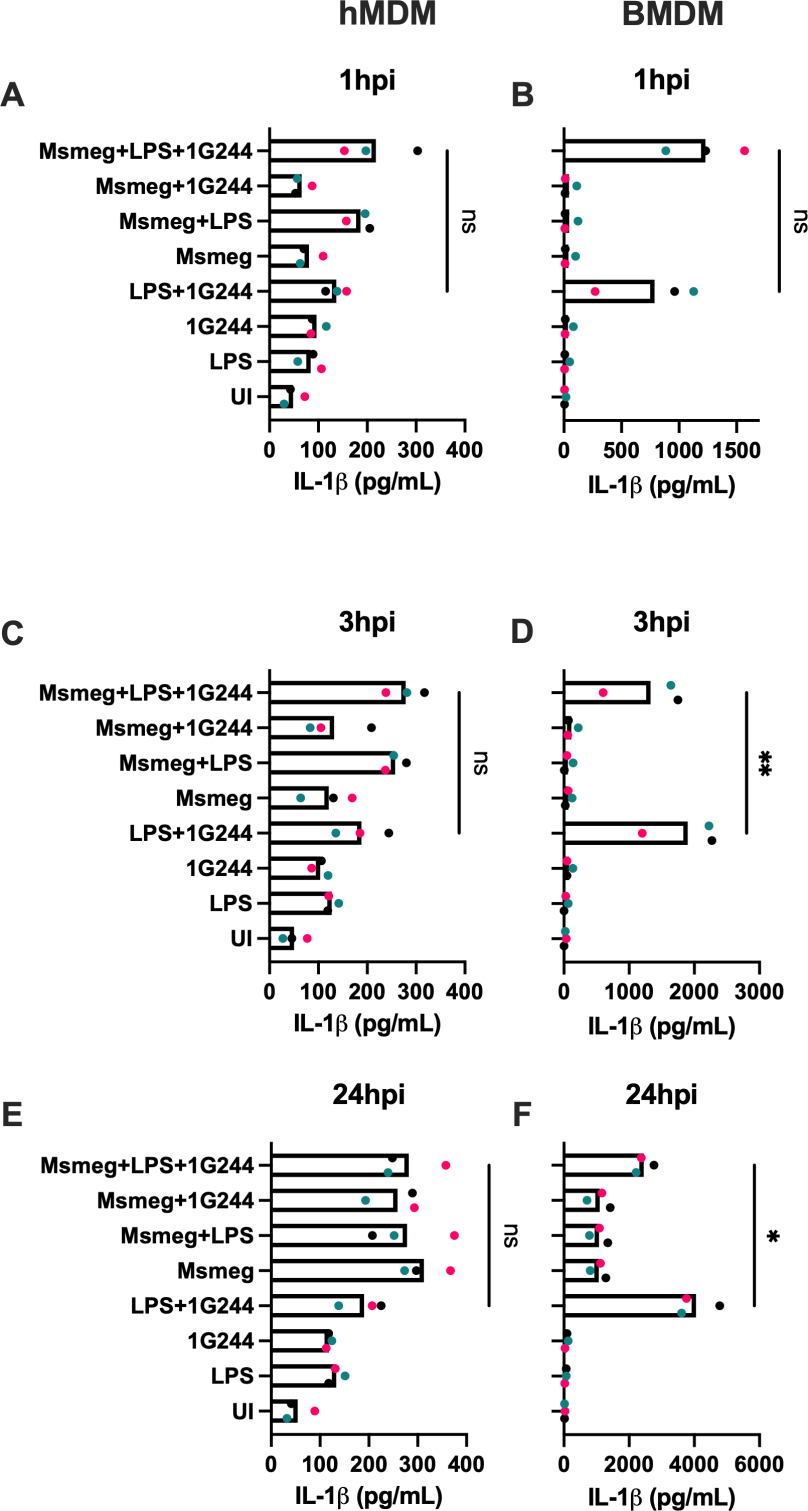
*M. smegmatis* fails to inhibit the NLRP1 inflammasome at 1, 3, and 24 h post-infection in human macrophages. hMDMs (left) and BMDMs (right) were infected with Msmeg at a multiplicity of infection (MOI) of 10 for 2 h. Indicated groups were treated with 0.5 µg/mL LPS for 2 h as well. Following infection/treatment, the indicated groups were treated with 1G244. At 1 (**A and B**), 3 (**C and D**), and 24 (**E and F**) h post-infection, IL-1β release was determined via ELISA. Statistical significance was determined by paired parametric *t*-tests. Independent biological replicates are shown. *P < 0.05; **P < 0.01; ns, not significant.

BMDMs and hMDMs were either infected with Msmeg for 2 h or left uninfected and treated with or without LPS. After infection, the cells were washed three times with PBS and treated with 1G244. At 1, 3, and 24 h, IL-1β was determined via ELISA. The cells treated with LPS + 1G244 were considered the positive control for NLRP1 activation. Msmeg failed to inhibit the NLRP1 inflammasome at all time points observed in hMDMs (Fig. 1A, C and E). Interestingly, in BMDMs, Msmeg-infected cells treated with LPS and 1G244 produced significantly less IL-1β at 3 and 24 h post-infection compared to treated uninfected cells (Fig. 1B, D and F). Cell death analysis showed no significant differences between the LPS + 1G244 and Msmeg + LPS + 1G244 groups at all time points observed (Fig. S2), leading us to conclude that Msmeg is unable to inhibit the NLRP1 inflammasome in hMDMs at the time points of interest. In contrast, there is some inhibitory activity at 3 and 24 h post-infection in BMDMs. Additionally, the IL-1β release between BMDMs and hMDMs is quite different, with BMDMs releasing significantly more IL-1β than hMDMs.

### *M. kansasii* does not inhibit the NLRP1 inflammasome at 1 and 24 h post-infection

**Fig 2:**
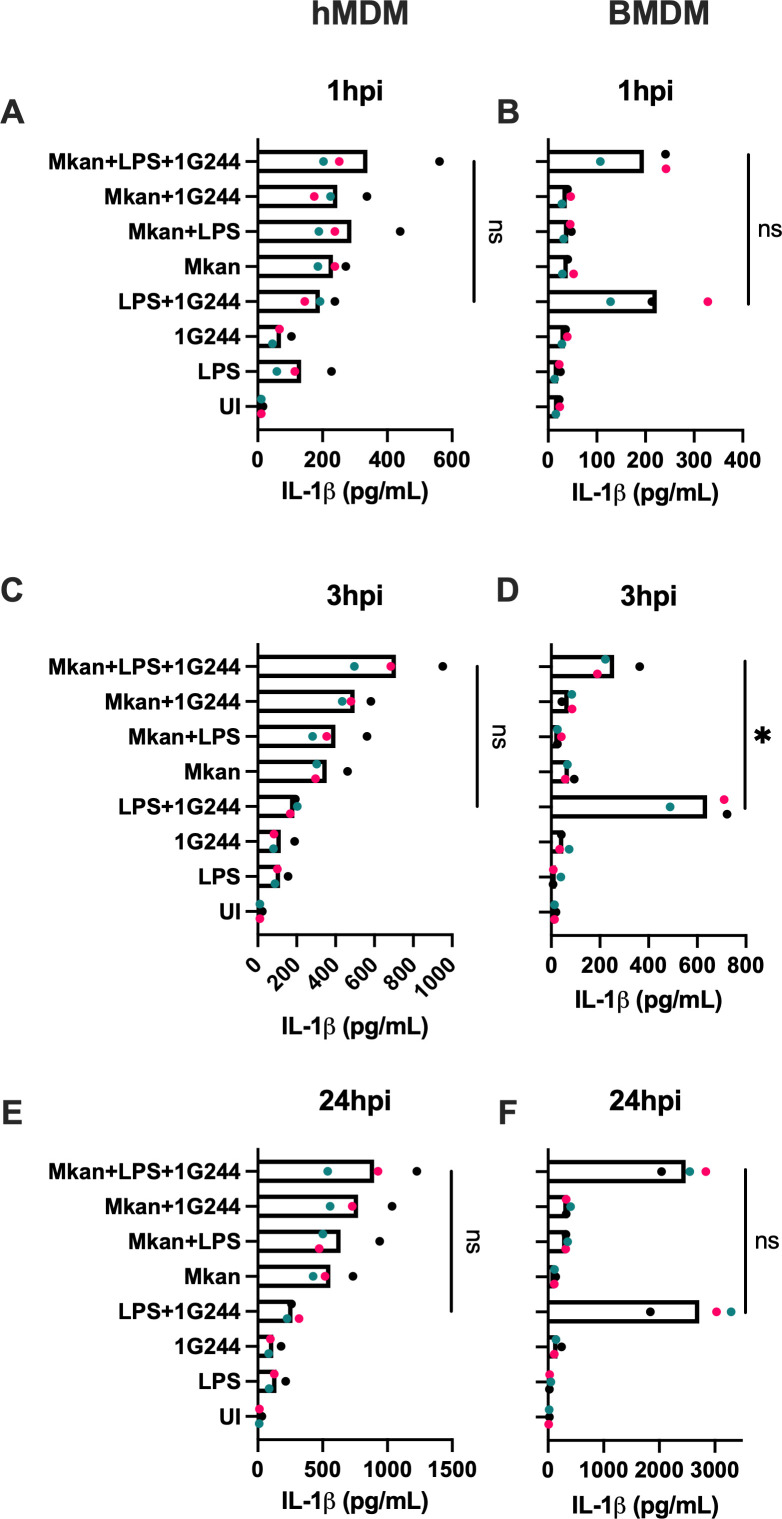
*M. kansasii* fails to inhibit the NLRP1 inflammasome at 1 and 24 h post-infection. HMDMs (left) and BMDMs (right) were infected with Mkan at an MOI of 10 for 4 h. Indicated groups were treated with 0.5 µg/mL LPS for 4 h. Following infection and/or LPS stimulation, the indicated groups were treated with 3 µM 1G244 for 1, 3, and 24 h. IL-1β release in hMDMs and BMDMs macrophages at 1 (**A and B**), 3 (**C and D**), and 24 (**E and F**) h post-infection as determined by ELISA. Statistical significance was determined by paired parametric *t*-tests, comparing LPS + 1G244 and Mkan + LPS + 1G244. Independent biological replicates are shown. *P < 0.05; ns, not significant.

Following the determination that Msmeg does not inhibit the NLRP1 inflammasome in hMDMs, we aimed to test whether Mkan behaves similarly. Mkan is a more virulent mycobacterial species, also closely related to Mtb and known to activate the NLRP3 inflammasome (7). HMDMs and BMDMs were treated with LPS and/or infected with Mkan for 4 h at an MOI of 10. Following infection, the cells were washed 3× with PBS, and the indicated groups were treated with 1G244. IL-1β release was observed at 1, 3, and 24 h post-infection (Fig. 2). At 3 h post-infection, BMDMs infected with Mkan and treated with 1G244 produce significantly less IL-1β than the LPS + 1G244-treated group, but not at 1 or 24 hpi (Fig. 2B, D and F). Further kinetic analysis on IL-1β production throughout the course of infection and treatment is required. There is no significant difference between the LPS + 1G244-treated cells and Mkan +LPS + 1G244 cells in hMDMs at all the observed time points. This indicates that there is no inhibition of the NLRP1 inflammasome by Mkan in hMDMs. Cell death analysis shows no significant difference between infected and noninfected hMDMs and BMDMs treated with LPS + 1G244 at 1 h post-infection (Fig. S3A and B). At 3 h post-infection, BMDMs infected with Mkan and treated with LPS and 1G244 showed significantly more cytotoxicity than the uninfected group treated with LPS and 1G244 (Fig. S3C and D). hMDMs showed minimal changes between the infected and uninfected groups at all time points of interest (Fig. S3A, C and E).

### *M. tuberculosis* does not inhibit the NLRP1 inflammasome at 3 and 24 h post-infection

**Fig 3:**
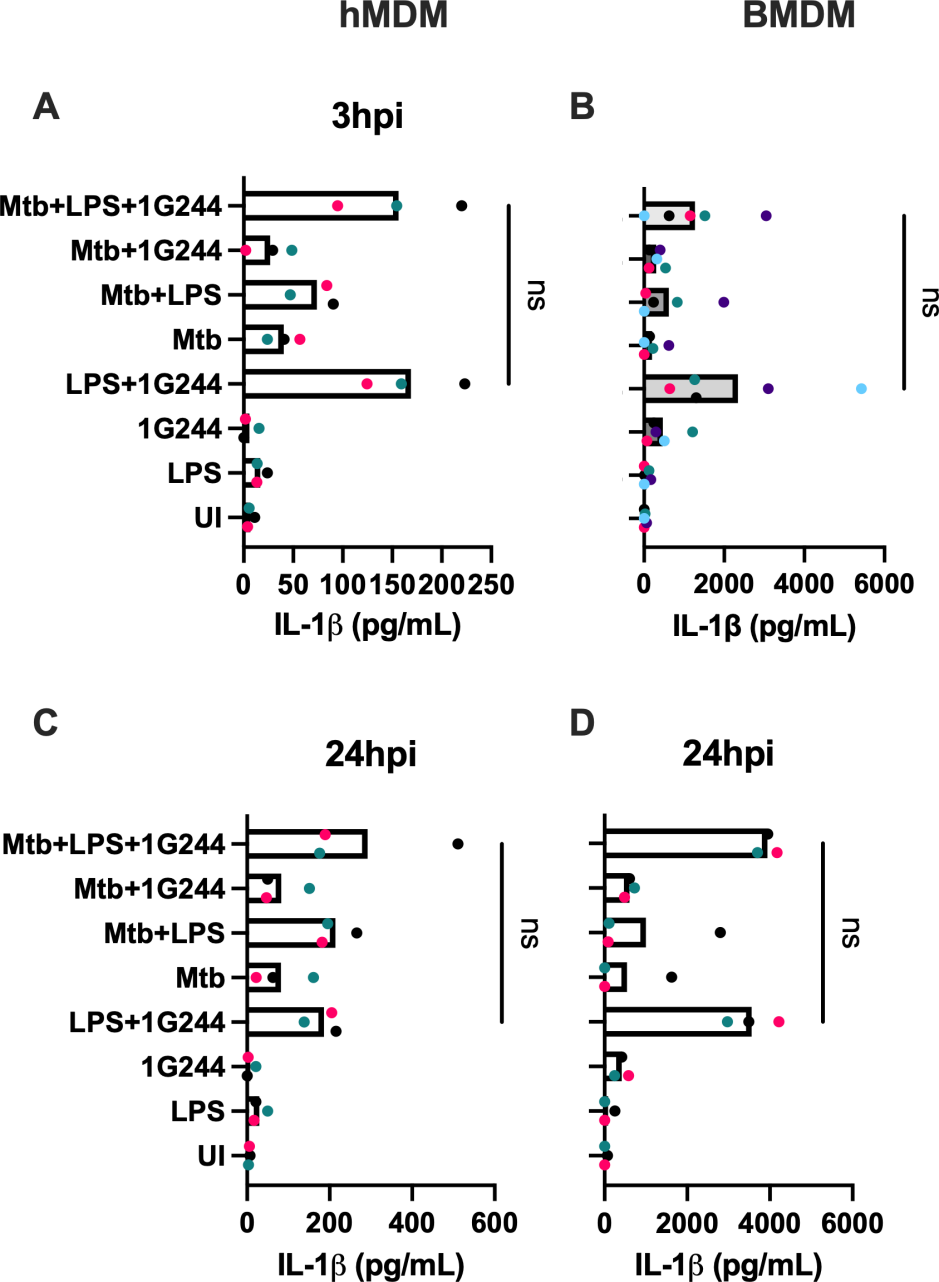
*M. tuberculosis* fails to inhibit the NLRP1 inflammasome at 3 and 24 h post-infection. HMDMs and BMDMs were infected with Mtb for 4 h at an MOI of 10. Indicated groups were also stimulated with 0.5 µg/mL of LPS for 4 h. Following infection/stimulation, the cells were washed three times with PBS. The indicated groups were then treated with 1G244, and supernatants were collected at 3 and 24 h for analysis. IL-1β release in hMDMs (**A and C**) and BMDMs (**B and D**) at 3 (**A and B**) and 24 (**C and D**) h post-infection as determined by ELISA. Statistical significance was determined by paired parametric *t*-tests, comparing LPS + 1G244 and Mtb + LPS + 1G244. Independent biological replicates are shown. Error bars represent the standard error of the mean. ns, not significant.

To analyze whether Mtb can inhibit the NLRP1 inflammasome, we tested 3 and 24 h time points. This was a departure from the experiments conducted with Msmeg and Mkan because of the slow growing nature of Mtb and previous empirical evidence that at 1 h post-infection, IL-1β release is minimal, making ELISA-based analysis unreliable (22, 27). hMDMs and BMDMs were infected with Mtb at an MOI of 10 and where indicated, simultaneously stimulated with LPS for 4 h. Subsequently, the cells were washed 3× with PBS and treated with 1G244. Supernatants were collected at 3 and 24 h post-infection. There is no significant difference in IL-1β release between the positive control and infected groups at all time points tested for both hMDMs and BMDMs (Fig. 3). Contrastingly, in BMDMs, infected cells treated with 1G244 and LPS show significantly more cell death than their noninfected counterparts (Fig. S4B and D). hMDMs show no significant difference in cell death between 1G244- and LPS-treated groups and their infected counterparts (Fig. S4A and C).

IL-1β-deficient mice show hyper-susceptibility to Mtb infection (18), demonstrating that it has a protective role in infection. Therefore, it can be surmised that the inhibition of the inflammasome, a major contributor to IL-1β release, is beneficial for pathogenesis of Mtb. It has previously been shown that Mtb can inhibit the NLRP3 through a variety of mechanisms (21, 22); however, it is unknown whether any other inflammasomes are inhibited as well. Overall, we have shown that Mtb does not inhibit the NLRP1. Interestingly, Msmeg and Mkan show some inhibitory activity against the NLRP1 inflammasome. The nature of this effect seems transitory in the case of Mkan but persists for Msmeg. This shows that inhibition is not necessarily correlated with virulence. It is important to note, however, that the higher-than-expected variance between biological replicates, especially for the 3 h time point of Mtb, makes it difficult to state definitively that no inhibition is occurring. To account for this, we have color-coded each individual biological replicate to better appreciate the overall trend between experimental groups.

### Mycobacteria fail to inhibit the pyrin inflammasome at 2 and 6 h post-infection

Rho GTPases are proteins that exist as a type of molecular switch for a large variety of processes within the cell, for example, cytoskeleton organization and immune cell migration (28). Pyrin specifically senses modifications in RhoA, which affect its binding to certain serine/threonine protein kinases. Virulence factors from pathogens can downregulate the activity of RhoA, and this disruption can induce the activation of the pyrin inflammasome in BMDMs. TcdB is a toxin released by *Clostridium difficile* that is known to downregulate RhoA and induce activation of the pyrin inflammasome (29). To determine the optimal dose of TcdB and LPS, a dose-response study was conducted where BMDMs were pretreated with LPS for 4 h, washed 3× with PBS, and then treated with TcdB toxin for 2 or 6 h (Fig. S5); 200 ng/mL of TcdB + 0.5 µg/mL of LPS was chosen as the optimal dose because it was able to elicit a robust IL-1β response at both 2 and 6 h post-infection (Fig. S5A and B).

**Fig 4:**
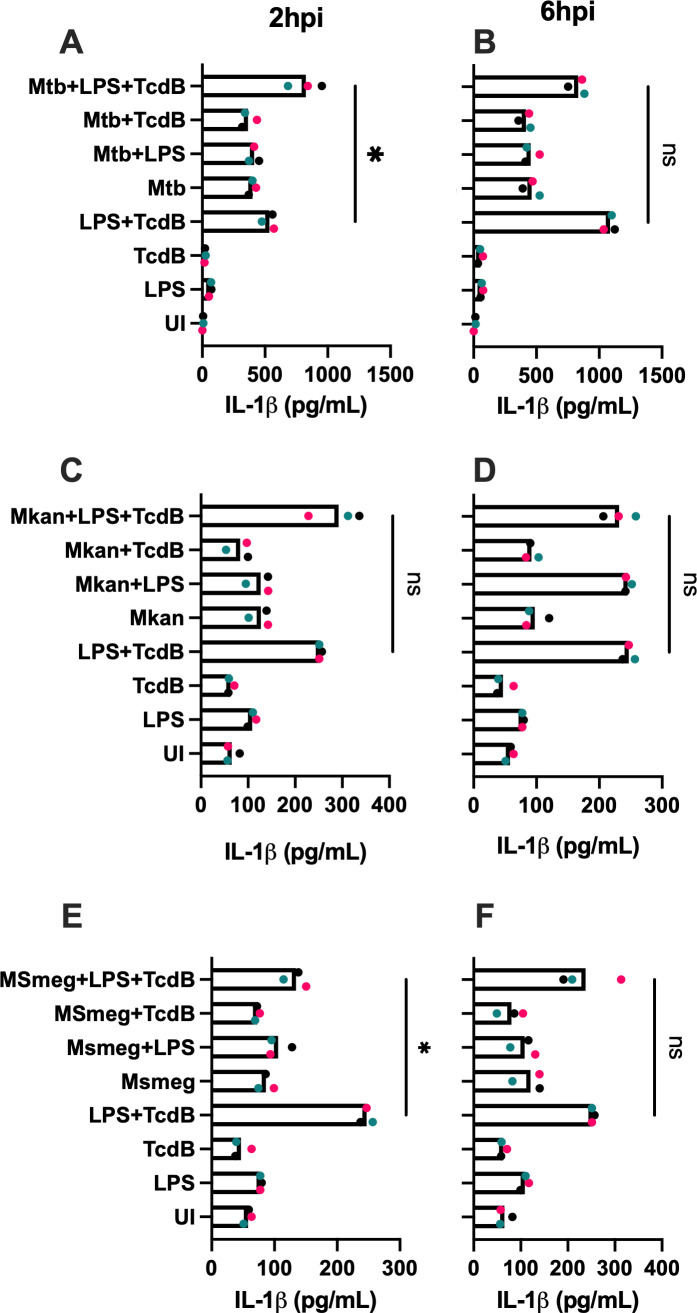
Mycobacteria fail to inhibit the pyrin inflammasome at 6 h post-infection. IL-1β release in BMDMs infected with Mtb (**A and B**), Mkan (**C and D**), and Msmeg (**E and F**) for 2 h at an MOI of 10 (Msmeg) or 4 h at an MOI of 10 (Mtb and Mkan). Indicated groups were stimulated with 0.5 µg/mL LPS alongside infection for 2 h (Msmeg) or 4 h (Mtb and Mkan). Following three PBS washes, cells were treated with TcdB. Supernatants were collected to determine whether IL-1β release at 2 and 6 h post-infection by ELISA. Statistical significance was determined by paired parametric *t*-tests, comparing LPS + TcdB and Mycobacteria + LPS + TcdB. Independent biological replicates are shown. *P < 0.05; ns, not significant.

To determine if the pyrin inflammasome is inhibited by mycobacteria, BMDMs were infected with Mtb, Mkan, or Msmeg and where indicated, also stimulated with LPS for 4 h. Following this stimulation/infection, cells were washed 3× with PBS and treated with TcdB for 2 and 6 h. Supernatants were collected and analyzed for IL-1β release and cytotoxicity. Mtb and Mkan fail to inhibit the pyrin inflammasome at both time points (Fig. 4A and B). Interestingly, Msmeg-infected cells treated with LPS and TcdB show significantly less IL-1β at 2 h post-infection than cells treated with LPS and TcdB alone (Fig. 4E). This indicates that some inhibition is occurring, the nature of which remains to be explored. Mtb-infected cells treated with LPS and TcdB show significantly more cell death than LPS- and TcdB-treated cells at 2 hpi, but this trend disappears by 6 h post-infection (Fig. S6A and B). No significant difference in cell death was observed between Mkan- and Msmeg-infected cells treated with LPS and TcdB and their noninfected counterparts at 2 or 6 hpi (Fig. S6C through F).

Further studies need to be conducted to determine the specific mechanisms of inhibition observed by Mkan and Msmeg at the indicated time points. Furthermore, the phenomena observed within the context of this study remain limited by our experimental setup. We chose to use IL-1β and LDH release as our main readout of inflammasome activation and/or pyroptosis, respectively. The use of more proximal readouts of inflammasome activity (e.g., GSDMD cleavage) may provide additional insights and might provide a higher resolution for observing potential inhibitory activity by the mycobacteria. Nevertheless, one could argue that if these differences do not lead to observable differences in IL-1β secretion or LDH release, their biological relevance would be minor. The role of IL-1β could be dependent on a variety of spatial and temporal factors, leading to beneficial or detrimental roles to the host. This would provide further understanding of the interplay between host and pathogen in nontuberculous mycobacteria, as well as elucidating the complicated role of IL-1β in infection. IL-1β is an important cytokine to ensure enough resistance to pathogenic infection while also preventing uncontrollable inflammatory pathology (30).

## MATERIALS AND METHODS

### Bacterial strains and growth conditions

*M. tuberculosis* (H37Rv), *M. kansasii* (ATCC1278), and *M. smegmatis* (mc^2^155) strains were grown in liquid Middlebrook 7H9 medium supplemented with 10% oleic acid-albumin-dextrose-catalase growth supplement (B12351), 0.2% glycerol, and 0.05% Tween 80.

### Cell culture and infections

Bone marrow cells were aseptically flushed from the femurs and tibias of wild-type (WT) C57BL/6J or S129 mice obtained from Jackson Laboratories. Bone marrow cells were cultured for 7 days in Dulbecco’s modified Eagle medium (DMEM) containing 10% heat-inactivated FBS, 20% L929 conditioned medium, and 1% penicillin/streptomycin at 37°C and 5% CO_2_ to allow differentiation into macrophages. After differentiation, BMDMs were harvested, plated into 96-well plates (5 × 10^4^ cells per well) in antibiotic-free BMDM media, and allowed to adhere and rest for 24 h just before the day of infection. On the day of infection, media were removed, and the cells were washed twice with pre-warmed 1× PBS. BMDMs were then infected with mycobacteria in DMEM supplemented with 10% FBS and 20% L929 conditioned media at an MOI of 10:1. Infected macrophages were maintained at 37°C in a humidified atmosphere with 5% CO_2_. After 4 (Mtb and Mkan) or 2 h (Msmeg) phagocytosis period, infected BMDMs were gently washed four times with pre-warmed 1× PBS before replacing with BMDM chase media containing DMEM supplemented with 10% FBS, 20% L929 conditioned media, and 10 μg/mL gentamicin.

Peripheral blood mononuclear cells (PBMCs) from healthy blood donors were isolated from leukopaks by using Ficoll density gradient centrifugation. Then, monocytes were isolated from PBMCs by CD14 MicroBeads (Miltenyi Biotec 130-118-906). Monocytes were differentiated into macrophages using 10 ng/mL hM-CSF (R&D Systems) for 7 days in RPMI1640 containing 5% off-clot human serum (Gemini), 1% penicillin/streptomycin at 37°C, and 5% CO_2_. hMDMs were then infected at day 7 at an MOI of 10:1 with mycobacteria in RPMI1640 supplemented with 10% human AB serum for either 4 (Mtb and Mkan) or 2 h (Msmeg).

### Cell culture treatments

BMDMs (5 × 10^4^) were seeded in 96-well plates in DMEM supplemented with 10% FBS and 20% L929 conditioned media. When required, the cells were treated with 0.5 µg/mL LPS (tlrl-3pelps) for 4 h and then washed 3× with pre-warmed PBS. Cells were then treated with 3 µM 1G244 (SML2247) or 200 ng/mL TcdB (6246-GT-020) for the time points of interest either alongside infection with Mtb, Mkan, Msmeg, or alone. Uninfected/untreated samples were used as control. Cell culture supernatants were harvested at the indicated time points and analyzed for IL-1 secretion by ELISA and for cytotoxicity by LDH release.

HMDMs (5 × 10^4^) were seeded in 96-well plates in RPMI1640 containing 5% off-clot human serum (Gemini). When required, the cells were treated with 1G244 or TcdB either alongside infection with Mtb, Mkan, Msmeg, or alone. Uninfected/untreated samples were used as a control. Cell culture supernatants were harvested at indicated time points and analyzed for IL-1β secretion by ELISA and cytotoxicity by LDH release.

### LDH cytotoxicity assay

Macrophage cell death was assessed by the release of LDH due to the loss of cellular membrane integrity. To measure LDH activity, 50 µL of cell culture supernatants from a 96-well plate were collected at indicated times, and LDH activity was measured using the CytoTox 96 Non-Radioactive Cytotoxicity Assay according to the manufacturer’s instructions (Promega, G1780).

### Cytokine ELISAs

Cell culture supernatants harvested from infected and treated cells were assayed for the levels of secreted IL-1 using the Human Total IL-1 DuoSet ELISA Kit (Bio-Techne, DY201) or the Mouse Total IL-1β DuoSet ELISA Kit (Bio-Techne, DY401) following the manufacturer’s instructions.

### Statistical analysis

GraphPad Prism software was used for graphing data and statistical analyses. Statistical significance was determined using a paired parametric *t*-test. Data were graphed so that each data point represents the mean of technical triplicate wells for one experiment, and all results are data from at least three independent biological replicates, unless noted otherwise. Differences were considered statistically significant if the *P* value was < 0.05.

## Data Availability

All data are contained within the article and its supplemental figures.

## SUPPLEMENTAL MATERIAL

Supplemental figures spectrum.04121-25-s0001.docx Fig. S1 to S6.

ASM does not own the copyrights to Supplemental Material that may be linked to, or accessed through, an article. The authors have granted ASM a non-exclusive, world-wide license to publish the Supplemental Material files. Please contact the corresponding author directly for reuse.
