# Supplementary material for: Mycobacterium tuberculosis does not inhibit NLRP1 and pyrin inflammasomes in human macrophages
Source: Microbiol Spectr. 2026 Jun 18;14(8):e04121-25. doi: 10.1128/spectrum.04121-25 (PMC13436130; doi:10.1128/spectrum.04121-25)
Supplement: Supplementary file 1 [file spectrum.04121-25-s0001.docx]

**Supplementary Figure 1:** Treatment of BMDMs and hMDMs with indicated concentrations of LPS and 1G244. BMDMs (A, B, C, D) and hMDMs (E, F, G, H) were stimulated with 0.5 µg/mL LPS for 4 hours. Cells were then washed three times with PBS and then treated with 1G244 and supernatants were collected at the indicated timepoints. IL-1β release was determined by ELISA and cell death was determined by LDH release.

**Supplementary Figure 2:** Cell Death as measured by LDH release indicates no significant difference in cytotoxicity between infected BMDMs and hMDMs treated with LPS and 1G244, and their uninfected counterparts at all time points of interest (A, B, C). Statistical significance determined by paired parametric t-tests, comparing LPS+1G244 and Msmeg+LPS+1G244. Shown are independent biological replicates. *P < 0.05, **P<0.01, ns, not significant.

**Supplementary Figure 3:**  Cell death as measured by LDH release shows no significant difference between BMDMs infected with Mkan and treated with 0.5 µg/mL LPS LPS and 3 µM 1G244 and their uninfected counterparts at 1, 24 hour post-infection (A, B, E, F). Statistical significance determined by paired parametric t-tests, comparing LPS+1G244 and Mkan+LPS+1G244. Shown are independent biological replicates. *P < 0.05, **P<0.01, ns, not significant.

**Supplementary Figure 4:**  Cell death, as measured by LDH, indicates no significant difference in cytotoxicity between groups in hMDMs at both time points (A, C). Infected BMDMs treated with 0.5 µg/mL LPS and 3 µM 1G244 show significantly more cell death than uninfected cells treated with LPS+1G244 (B, D). Statistical significance determined by paired parametric t-tests, comparing LPS+1G244 and Mtb+LPS+1G244. Shown are independent biological replicates. *P < 0.05, **P<0.01, ns, not significant.

**Supplementary Figure 5:** Treatment of BMDMs with LPS and TcdB induces IL-1β.

BMDMs were stimulated with 0.5 µg/mL of LPS for 4 h then washed 3x with PBS. BMDMs were then treated with TcdB at the indicated concentrations. Supernatants were collected at 2 and 6 hours post TcdB treatment for IL-1β release analysis and cell death analysis.

**Supplementary Figure 6:** Cell death, as measured by LDH, indicates no significant difference in cytotoxicity between groups in BMDMs infected with Mkan and Msmeg at both time points (C, D, E, F). BMDMs infected with Mtb show significantly more cytotoxicity than BMDMs treated with 0.5 µg/mL LPS and 200 ng/mL of TcdB at 2 hours post infection (A) however this difference disappears by 6 hours post infection (B). Statistical significance determined by paired parametric t-tests, comparing LPS+1G244 and Mtb+LPS+1G244. Shown are independent biological replicates. *P < 0.05, **P<0.01, ns, not significant.
